# Assessment of Algorithms for Inferring Positional Weight Matrix Motifs of Transcription Factor Binding Sites Using Protein Binding Microarray Data

**DOI:** 10.1371/journal.pone.0046145

**Published:** 2012-09-28

**Authors:** Yaron Orenstein, Chaim Linhart, Ron Shamir

**Affiliations:** Blavatnik School of Computer Science, Tel-Aviv University, Tel-Aviv, Israel; University of Georgia, United States of America

## Abstract

The new technology of protein binding microarrays (PBMs) allows simultaneous measurement of the binding intensities of a transcription factor to tens of thousands of synthetic double-stranded DNA probes, covering all possible 10-mers. A key computational challenge is inferring the binding motif from these data. We present a systematic comparison of four methods developed specifically for reconstructing a binding site motif represented as a positional weight matrix from PBM data. The reconstructed motifs were evaluated in terms of three criteria: concordance with reference motifs from the literature and ability to predict *in vivo* and *in vitro* bindings. The evaluation encompassed over 200 transcription factors and some 300 assays. The results show a tradeoff between how the methods perform according to the different criteria, and a dichotomy of method types. Algorithms that construct motifs with low information content predict PBM probe ranking more faithfully, while methods that produce highly informative motifs match reference motifs better. Interestingly, in predicting high-affinity binding, all methods give far poorer results for *in vivo* assays compared to *in vitro* assays.

## Introduction

Understanding gene regulation is a fundamental problem in biological research. A principal way to regulate gene expression in the cell is via transcription, which is governed primarily by transcription factors (TFs). A TF is a protein that binds to the promoter region of a gene at specific sequences, called TF binding sites (TFBSs). The binding of one or several TFs enables or impedes the transcription of the gene. A TF binds to similar short nucleotide sequences at different affinities. Finding these cis-regulatory elements and modeling the affinity of TF binding to them is a central challenge in understanding gene regulation.

The most common computational model for describing a TFBS motif is a position weight matrix (PWM) [1]. The TFBS is represented by a 4×*k* matrix, where *k* is the motif length. Each column contains four probabilities, representing the nucleotide frequencies at that position. This relatively simple model is highly popular since it is compact, effective and easy to interpret.

New technologies have enabled comprehensive mapping of protein-DNA binding affinities. The main technology to measure *in vivo* protein occupancy is chromatin immunoprecipitation (ChIP). In the ChIP-chip method, the protein-bound DNA segments are hybridized to a pre-designed microarray [2], whereas the ChIP-seq method uses deep sequencing to read the bound DNA segments [3]. A recent promising technology in this field is the protein binding microarray (PBM) [4]. This microarray contains ∼41,000 synthesized, 60 bp-long double-stranded DNA probes, each containing 36 bp of unique sequence, designed so that every possible 10-mer is contained in exactly one probe sequence. A single *in vitro* experiment measures the binding intensity profile of a specific TF to each probe, thereby providing complete coverage of the binding affinity of the TF to all possible 10-mers. Often, two experiments with different array designs are performed with the same TF, providing *paired* profiles.

Numerous computational methods for finding a motif in a target set of promoters have been developed over the last two decades [5]–[7]. Predicting binding sites based on PBM data is different: the experimental data are much more comprehensive, covering all possible 10-mers, but are generated *in vitro* and in a high-throughput (and hence noisy) fashion. Therefore, several methods were recently developed specifically for identifying TFBS motifs from PBM profiles. Here we compare methods that represent the motifs as PWMs. We do not include methods that use more complex models [8], since we choose to focus on simpler, more compact models.

In this paper we present a systematic comparison of four algorithms for identifying TFBS motifs from PBM profiles: Seed-and-Wobble (SW) [4], RankMotif++ (RM) [9], BEEML-PBM (BE) [10] and the algorithm Amadeus-PBM (AM) introduced here (see **Table 1**). In 2005, a systematic comparison of computational methods for motif discovery in promoters clarified some of the issues and the difficulties in that domain, and led to progress in that research area [11]. We hope that our study will have a similar effect regarding methods for analyzing PBM data.

**Table 1:** Properties of the tested methods.

Program	Operating principle	Reference
Seed-and-Wobble	Ranks all 8-mers according to Wilcoxon-Mann-Whitney rank-sum score. The top scoring 8-mer is used as a seed, its positions are “wobbled” and its length is extended in order to improve match to the data. http://the_brain.bwh.harvard.edu/PBMAnalysisSuite/index.html	[4]
RankMotif++	Aims to predict the ranking of the probes according to their binding intensity. Maximizes the likelihood of the ranking function, using the three top 7-mers as seeds. http://morrislab.med.utoronto.ca/software.html	[9]
BEEML-PBM	Estimates the position and background biases from the data, then optimizes the parameters of a binding energy model using BEEML algorithm, explicitly taking the biases into account. http://stormo.wustl.edu/beeml/	[10]
Amadeus-PBM	Seeks enriched PWMs in 1000 top ranking 9-mers compared to the background set of all 9-mers, using Amadeus motif finding algorithm. http://acgt.cs.tau.ac.il/amadeus//	Described here

## Results

### Concordance with SELEX-based reference motifs from the literature

We used each method to find motifs using PBM data, and compared the results to previously reported motifs for the same TFs, obtained using independent experiments. Each motif was learned using the data from two paired experiments performed with the same TF. For each TF, we measured the distance between the PBM-based PWM to the PWM of the same TF as published in JASPAR [12]. For this test we used all mouse PBM datasets from the SCI09 study [13], [14] that had a corresponding PWM in JASPAR, excluding those for which the JASPAR PWMs were constructed using PBM data. This set contained 58 PWMs. Most were constructed based on *in vitro* SELEX experiments, which are still the main source of TF motifs.

The AM PWMs were the most similar to JASPAR, with average Euclidean distance (± estimated standard deviation) 0.178±0.11. The average for SW was 0.193±0.1, for RM was 0.21±0.09, and for BE was 0.227±0.1 (**Table 2**). The difference between AM and SW was not significant (p = 0.17, Wilcoxon rank-sum test) and both were significantly better than RM and BE (p = 0.001 and p = 0.0005 compared to AM, respectively).

**Table 2:** Summary of the comparison. Boldface indicates significantly better performance than the other methods (including equal top performance).

	Similarity to reference motifs	*In vitro* binding prediction			*In vivo* binding prediction			Running time
	Average Euclidean distance	Spearman rank coefficient	Sensitivity at 1% FP	AUC	Spearman rank coefficient	Sensitivity at 1% FP	AUC	Seconds
**AM**	**0.178**	0.27	0.342	0.876	**0.152**	0.089	**0.653**	**30**
**SW**	**0.193**	0.244	0.305	0.866	**0.145**	**0.118**	**0.659**	7200
**RM**	0.21	0.264	0.295	0.881	**0.158**	0.092	**0.655**	3600
**BE**	0.227	**0.308**	**0.411**	**0.891**	**0.146**	0.084	**0.665**	900

**Figure 1:**
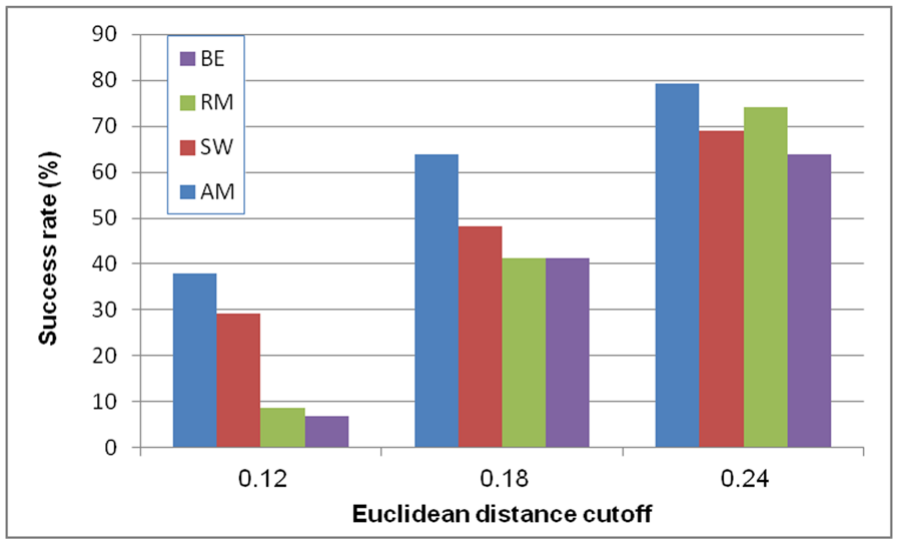
Similarity to experimentally established PWMs. For 58 TFs, we compared the motifs produced from their PBM profiles by each method, to the known motif from JASPAR database. Distance was measured using Euclidean distance. Three distance cutoffs were used, and the fraction of recovered motifs with distance below the cutoff is the success rate. BE: BEEML-PBM, RM: RankMotif++, SW: Seed-and-Wobble, AM: Amadeus-PBM, JR: JASPAR.

We then focused on high-quality predictions of the four methods. We say that a motif is successfully recovered by a method if the Euclidean distance of the predicted PWM from the reference PWM is below a predetermined cutoff. As in [15], we used three cutoffs for the distance. AM attained a higher success rate using all cutoffs (**Figure 1**). A similar comparison of mouse motifs in TRANSFAC [16] and yeast motifs in ScerTF [17], and a parallel comparison, using p-value for the significance of the similarity [18], showed a similar advantage to AM (**Figure S1**).

**Figure 2:**
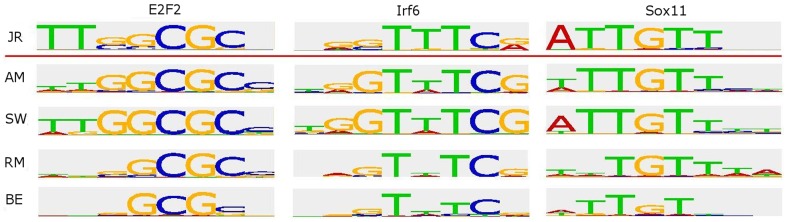
Examples of generated motifs. The figure shows examples of the motifs produced by each method and the corresponding JASPAR motif. For three proteins, the PWM logos produced by each method and the experimentally and independently established motif in the JASPAR database are shown. AM was trained on motif length 8, while for BE, RM and SW only the most informative contiguous positions were kept. We chose TFs whose motifs had information content most similar to the averages of the different methods.

Visual inspection suggested that the PWMs produced by AM and SW are easier to interpret and look distinct in logo format (**Figure 2**). To quantify this observation, we calculated the average information content for each PWM (see **Methods S1**). Averaged over the PWMs computed from all 115 available paired mouse PBM sets, the information scores for the raw PWMs were 1.03, 0.61, 0.42 and 0.53 bits for AM, SW, RM and BE, respectively, with AM scoring significantly higher (p<10^−15^, Wilcoxon rank-sum test). After trimming the PWMs to discard flanking positions with low information, the information averages were 1.03, 1.09, 0.54 and 0.61 bits, respectively (p = 1.2·10^−7^ when comparing SW to AM and <10^−15^ when comparing AM and SW to RM and BE). The full comparison results are available in **Table S1**.

### Predicting *in vitro* binding intensities

Next, we tested the prediction of binding intensities by the four methods on 115 pairs of mouse PBM profiles [13], [14] following the procedure in [9]. Each method learned a PWM according to one PBM experiment; this PWM was used to rank the probes of its paired array. The goal was to correctly rank the positive probes, i.e. those with highest affinity measurements. The set of positive probes (denoted 4σ, see **Methods S1**) contained an average of 912 probes per array. We also evaluated larger sets of positive probes using more permissive cutoffs (denoted 3σ, 2σ and 1σ; an average of 1580, 3215 and 8224 probes per array, respectively).

**Figure 3:**
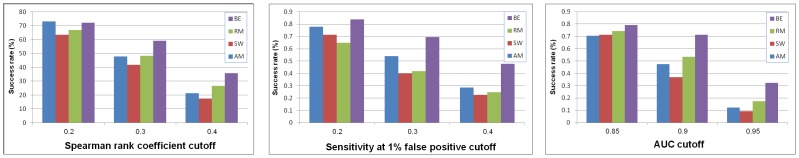
Success rates in probe ranking of a paired PBM. For each TF and method, the PWM was learned using one array and used to infer probe intensity ranking in its paired array. Ranking was gauged on a set of top positive probes (4σ set) according to three measures: Spearman rank coefficient, sensitivity at 1% false positive and AUC (see **Methods S1** for all mathematical terms). For each quality measure, three distance cutoffs were used, and the fraction of TFs with score equal or better to the cutoff is the success rate. The results show the success rate over 230 samples (115 paired arrays).

**Figure 4:**
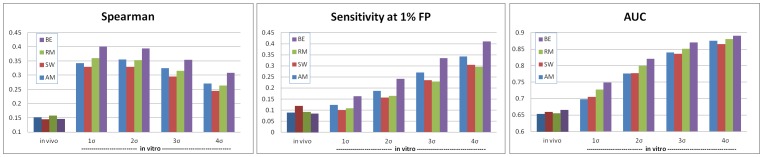
Quality of binding prediction for *in vivo* and *in vitro* data of different sizes. For each of the four algorithms, the quality of the motifs inferred from PBMs in ranking the top binding probes as measured *in vivo* (by ChIP-chip experiments) and *in vitro* (by PBMs) was evaluated. The *in vivo* test included 69 yeast ChIP-chip experiments data (with an average of 61 promoters per experiment). The *in vitro* test included 230 mouse PBMs covering 115 TFs, and used several definitions for the sets of top binding promoter sequences (4σ to 1σ, with averages of 912, 1580, 3215 and 8224 top probes, respectively, see text). Ranking quality was measured by the Spearman rank coefficient, the sensitivity at 1% false positive (FP) and the area under the ROC curve (AUC) (see **Methods S1**). The average ranking quality is reported in each case.

When testing on 4σ top probes set (**Table 2** and **Figure 3**), BE had significantly best Spearman and AUC scores (p<0.0025, Wilcoxon rank-sum test), while AM and RM were essentially equal (p = 0.41 and p = 0.44, respectively), and significantly better than SW (p<10^−4^). Using the sensitivity measure, BE was again best (p<10^−15^), AM second best (p = 3.8·10^−6^ compared to SW), and RM and SW were roughly the same (p = 0.18). Hence, BE showed consistently best performance in all three measures, followed by AM. Interestingly, BE gave the poorest AUC and Spearman scores on a few samples. On larger probe sets (**Figure 4**), BE performed best, followed by RM. The AUC and sensitivity criteria deteriorated for all methods, as expected due to the increasing difficulty in ranking lower-affinity probes. The Spearman score improvement results from its bias to larger sets, so it is more meaningful for comparison of sets of similar sizes. Full results are available in **Table S2**.

### Predicting *in vivo* binding intensities

Since PBM and SELEX are *in vitro* assays, which may introduce biases, we also tested the methods' abilities to predict binding intensities for *in vivo* experiments. Our evaluation included ChIP-chip datasets of 32 yeast TFs (69 experiments) that had also PBM profiles [19], [20]. A PWM learned according to the profiles of both PBMs (when available) is tested against the data from a ChIP-chip experiment. To evaluate the prediction on the high intensity promoters, where binding is expected to be strongest, we used the positive promoter set as those with reported p-values below 0.001.

**Figure 5:**
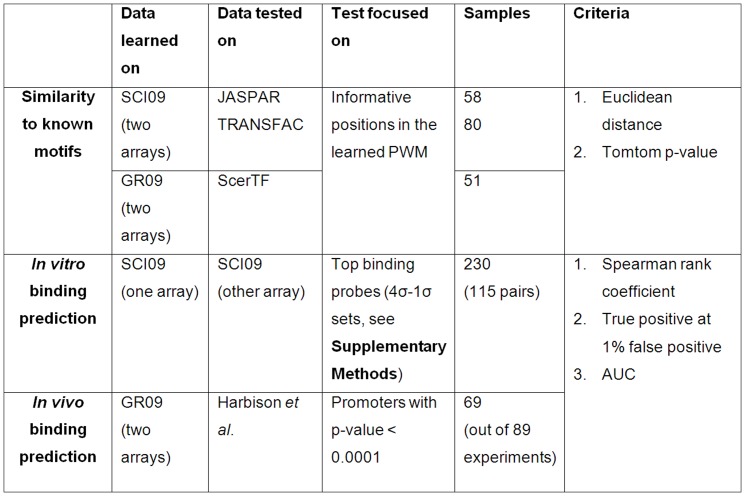
Test data and evaluation criteria. The table lists the data and evaluation criteria used in each benchmark.

All methods performed quite similarly on the AUC and Spearman rank coefficient criteria (**Figure 5**). Using the sensitivity measure, SW was better than the other three (p<0.02), AM and BE were roughly the same (p = 0.39) and significantly better than RM (p<0.04). Hence, SW showed consistently best performance in all three measures, while AM and BE were second best (**Table 2** and **Figure 4**). Full results are available in **Table S3**.

### Running times

We ran each method on the same 10 examples using a single core of an Intel® Xeon® CPU E5410 @ 2.33 GHz, with 6 MB of cache and 16 GB of memory. On average, AM runs for 30 seconds (including pre-processing), while BE, RM and SW run for about 15 minutes, one hour and more than two hours, respectively (**Table 2**). BE currently uses SW results as seeds, thus SW's running time should be added to the total running time of BE. Hence, AM provides a speedup by a factor of 30–200.

### Similarity between the algorithms

**Figure 6:**
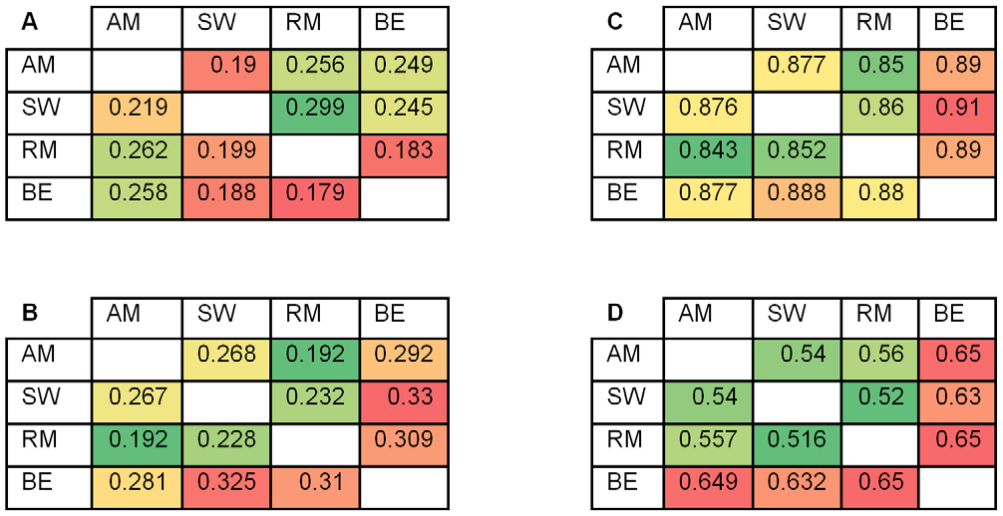
Similarity between methods. (A) For each pair of methods, the Euclidean distance between the PWMs of the two methods is reported. Before the comparison, the column method's PWM is trimmed to eight most informative contiguous positions. (B–D) ranking based comparisons. For each pair of methods, the probe ranking defined according to the column's method is used as reference, and the ranking of the row's method is evaluated using AUC (B) and sensitivity at 1% false positive (C). In (D), for each pair of methods, the 4σ positive sets of the paired PBM are first ranked by each method, and the Spearman rank coefficient of those rankings is computed. In all tables, the average over 230 PBM experiments is reported. Red colour corresponds to greater similarity.

We evaluated the similarity between the PWMs produced by the four algorithms (**Figure 6A**). In terms of PWM distance, the pairs AM/SW and RM/BE were more similar than others. Note that the comparison is not symmetrical, since it uses the eight most informative contiguous positions in the first PWM (corresponding to a column in the table). Large asymmetries (e.g., SW-RM and RM-SW) reflect the fact that these positions are not clearly detectable in RM and BE PWMs (see also **Figure 2**). On average, the distance between PWMs from different methods is similar to the distance between these and the reference PWMs (**Table 2**).

We also compared the probe ranking that the PWMs of the different algorithms induce (**Figure 6B–D**). We used a PWM inferred by one algorithm on a PBM to rank the probe set of the paired PBM, and measured sensitivity and AUC for these probes ranking produced by another algorithm. Results tended to show more symmetry, with pairs involving BE obtaining best scores, in agreement with the good performance of BE in ranking (**Figure 3** and **Table 2**). Additionally, we focused on rankings of the 4σ probe set and compared them using Spearman rank coefficient. PWMs inferred by two algorithms on a PBM to rank the 4σ probe set of the paired PBM, and compared the two rankings using Spearman score. Again pairs with BE got the highest scores, and remarkably, all pair scores were much higher than their similarity scores to original binding intensities (Spearman rank coefficient, 0.5–0.6 compared to 0.24–0.31, respectively).

## Discussion

We have described an assessment of four tools for extracting binding site motifs from PBM data. All four methods report their results in the form of a positional weight matrix (PWM). **Table 2** summarizes the comparison. All tools were run with their recommended default parameters; tuning the parameters could improve the results of some methods and affect the relative ranking in our test criteria.

The reference motifs stored in databases are strongly dependent on experimental sources. Most TRANSFAC and JASPAR motifs that we used were created based on SELEX, an *in vitro* assay of limited accuracy and throughput. Still, the relative performance of the methods was essentially the same when tested on three different databases of two species, which indicates robustness of our conclusions.

The best results in similarity of reference mouse motifs to predicted motifs from PBMs (**Figure 1**) were comparable to the similarity of reference metazoan motifs to predicted motifs obtained using a state-of-the-art motif finder that uses promoter sequences [15]. On one hand, PBM profiles cover the spectrum of possible sequences more comprehensively. On the other hand, they include only relatively short motifs. To conclude, no clear winner has yet emerged between PBM technology and traditional motif finding methods in finding PWMs that are closest to reference motifs.

When using binding intensities of one PBM as input and predicting the ranking of probe intensities of another array for the same TF, BE showed best performance. When using PBM binding intensities to predict ranking of promoter intensities in a ChIP-chip experiment for the same TF, SW performed best. We note that there is still only a modest number of TFs with data from both ChIP-chip and PBM; a larger benchmark for *in vivo* prediction, containing also TF binding in metazoans, is needed.

The performance results can be explained by the different goals of the algorithms. RM was designed to optimally rank all probes, so it tries to capture both high-affinity and low-affinity binding information. This explains why it performs less accurately when analyzing the top-binding probes but performs better on very large positive sets (**Figure 4**). The same applies to BE. The inclusion of information from low-intensity binding yields better ranking of low-affinity binding probes, but creates PWMs with lower information content (**Figure 2**). In contrast, AM was designed to identify specific binding motifs; it trains only on the 1000 top-binding 9-mers, and so it only uses information on the specific binding of the protein. Interestingly, SW is best for *in vivo* binding, hinting that longer motifs with a stringent core might be better for this data.

The comparison of the prediction results for *in vitro* and *in vivo* data (**Figure 4**) is striking: The quality of the results is much poorer on *in vivo* data, according to all evaluation criteria (similar results were reported in [21]). This is in spite of the fact that the *in vivo* data consisted of yeast motifs, which are easier to find than mice motifs [5], [15]. There can be several explanations of this finding:

1. The length of the probes on the PBM (36 bp) is much shorter than the whole yeast promoters targeted by ChIP-chip (an average of 474 bp). As a result, scoring and ranking yeast promoters is harder.
2. Biases caused by the PBM technology lead to systematic distortion in the reconstructed motifs, compared to *in vivo* motifs. If this is the case, revealing and correcting these biases is essential for using the motifs for *in vivo* analysis.
3. The methods tailored specifically for PBMs may overfit this type of data.
4. The complexity of *in vivo* assays distorts the raw binding signals, which look more like the PBM-based motifs in a cleaner *in vitro* environment.

One interesting phenomenon we encountered was secondary motifs: For some PBMs, SW and AM identified a second, completely different motif in addition to the primary one (**Figure S2**). This phenomenon was first reported in [14]. Agius *et al.* suggested that the secondary binding motifs arise as an artefact of the PBM experiment [8]. Zhao and Stormo suggested that secondary motifs are a result of a biased analysis of the PBM data [10], but Morris *et al.* challenge this conclusion [22]. We tested the benefit of using primary and secondary motifs discovered by SW for *in vitro* binding prediction. While there was a significant improvement in performance, it was still worse than BE (data not shown). Jauch et al. recently obtained a crystal structure of the TF Sox4 domain bound to DNA and concluded that two positions in the binding motif are dependent [23]. Such dependency can be manifested by two PWM motifs. Indeed, SW and to some extent AM recover two motifs that reflect this dependence (**Figure S3**). We agree with the conclusion in [21] that more matching PBM and *in vivo* datasets are needed in order to shed more light on this phenomenon.

An interesting insight arises from the comparison of the methods (**Figure 6D**). In terms of the Spearman score of probe ranking, all methods are much more similar to each other than to the true binding intensities. This suggests that all methods capture similar information, while missing other pertinent effects (e.g., background or technological biases). On the other hand, predicting the top probes of another method was harder than finding true positive probes (**Figure 6D**). Overall, BE had highest pairwise ranking-based scores, concordant with our conclusion that it predicts true binding best (**Table 2**). In terms of distance between PWMs, higher similarities between AM and SW, and between BE and RM, reflect the observation that the former pair produce clear, stringent motifs, while the latter generate more variable, ranking-oriented motifs.

Protein-DNA interactions can occur in a broad range of intensities, and involve both specific and low-affinity (less specific) binding. PBM data enable analysis of the full spectrum of DNA binding affinities of a TF. The binding specificity of a protein can be represented using various models, which differ in expressiveness, compactness, redundancy and interpretability. Our analysis suggests that a PWM models the specific *in vitro* binding quite accurately, obtaining an average AUC of 0.9 on the top probes. The fact that results of all methods tend to deteriorate as the positive sets grow (**Figure 4**), and the success of more complex models in ranking [8] suggest that less specific binding may be better captured by other models. The lower success of all methods in predicting *in vivo* binding questions the transformability of PBM-based results to the *in vivo* domain. Deeper analyses using more data are required on this point.

Our study gauged performance using three criteria: similarity to reference literature motifs, and ability to rank *in vitro* and *in vivo* bindings. The tested methods show a tradeoff between ranking quality and motif similarity. Degenerate motifs are better at *in vitro* binding prediction at the cost of lower information content and similarity to literature motifs. Potential improvement may be achieved by novel methods that strive to optimize both criteria simultaneously.

## Materials and Methods

### Algorithms

We compared four algorithms: Seed-and-Wobble (SW) [4], RankMotif++ (RM) [9], BEEML-PBM (BE) [10] and Amadeus-PBM (AM), a new algorithm presented here (see **Methods S1**). The computational approaches of the algorithms are summarized in **Table 1**. Software for BE, RM and SW was downloaded from the authors' websites and run using the default parameters. The full details are in **Methods S1**.

### PBM data

We downloaded PBM data from UniPROBE [13]. This database contains, for each TF, paired probe intensity profiles measured on two different arrays. We used the SCI09 dataset, which contains paired profiles of 115 mouse proteins [13], [14], and the GR09 dataset, which contains profiles of 89 yeast TFs [20] (**Figure 5**).

### Reference PWM data

To compare predicted PWMs to experimentally obtained PWMs, we used three databases of reference PWMs: JASPAR [12] and TRANSFAC [16] for mouse motifs and the new yeast motif database ScerTF [17] (**Figure 5**). We included in the comparison only reference PWMs that were produced without using PBM data.

### ChIP-chip data

We downloaded the ChIP-chip data for yeast TFs from Harbison *et al.* [19]. These data provide large-scale *in vivo* binding for many TFs. Our test used 69 experiments (32 TFs) that had PBM profiles in UniPROBE as well as ChIP-chip measurements.

### Comparison and evaluation

We tested the quality of PWMs produced by each method in three ways: by comparison to reference PWMs from the literature (mostly SELEX-based), by their accuracy in predicting *in vitro* binding in PBMs, and by their accuracy in predicting *in vivo* binding as measured by ChIP-chip. In addition, we evaluated how similar the methods are in a pairwise comparison using the same criteria.

To compare a predicted PWM to a reference one, the Euclidean distance between the two PWMs was calculated, as in [15] (for a description of all evaluation criteria see **Methods S1**). The information content of each matrix was also measured in order to evaluate its degeneracy. Each algorithm was trained using the data from both arrays for the same TF. PWMs were also compared using the Tomtom algorithm [24].

For testing the quality of *in vitro* binding prediction, we followed the method of [9]. Since two (paired) binding profiles were available for each TF, a PWM was trained on one profile (the “training array”) and used to rank the probes in the other profile (the “test array”). Given a PWM, the probes of the test array were ranked using the sum occupancy score (see **Methods S1**). This ranking was compared to the measured ranking of the probes in the test array according to three criteria: Spearman rank coefficient, sensitivity at 1% false positive rate and area under the ROC curve (AUC) (see **Methods S1** for all definitions). The comparison was done on the probes that showed high binding intensity in the test array (the positive probe set [9]).

To test the quality of *in vivo* binding predictions, we used similar criteria. For each TF, we trained each method using both paired binding profiles (when available) and tested how well the method predicts the ranking of the strongest bound yeast promoters (see **Methods S1**). Predicted and experimental rankings were compared using the same three criteria.

In computing similarity between different methods, we used four criteria. First, we measured the distance between the PWMs inferred by each method. Second, for each method, using the PWM learned on one array, we ranked the set of positive probes in the paired array, and then measured the Spearman rank coefficient between the rankings of each two methods. Third and fourth, we used one method to rank the probes of the paired array, and tested the prediction of the other method using sensitivity at 1% false positive and AUC (see **Methods S1** for computational details).

### Statistical significance of the comparison

For each comparison we evaluated its significance using the Wilcoxon rank-sum test [25]. Since the gauged measurements do not distribute normally, we used a non-parametric statistical test.

## Supporting Information

Figure S1 **Similarity to experimentally established PWMs.** (A) TRANSFAC motifs. For 80 proteins available in TRANSFAC we compared the motifs produced from their PBM data by each of the tested methods to the motif available in TRANSFAC. Distance was measured using Euclidean distance. Three distance cutoffs were used, 0.12, 0.18 and 0.24, and the fraction of recovered motifs with distance below the cutoff is the success rate. (B): ScerTF motifs. The same tests on 51 motifs from the ScerTF database. AM: Amadeus-PBM; SW: Seed&Wobble; RM: Rankmotif++; BE: BEEML-PBM.
(TIF)

Figure S2 **Shadow motifs.** Examples of the primary and secondary motifs found by Amadeus for Pou2f3 (A) and Sox1 (B). p-values for the motif enrichment (hypergeometric score) are indicated above each motif. Note that even the second ranked motifs obtain extremely high significance.
(TIF)

Figure S3 **Sox4 primary and secondary motifs as found by Seed-and-Wobble (SW) and Amadeus-PBM (AM).** Jauch et al. reported two motifs: CTTTGTT and AATTGTT (23). (A) The two top motifs recovered by AM. The first motif of Jauch et al. was recovered correctly; the second was partially recovered. (B) The two top motifs recovered by SW. Both motifs from Jauch et al. were inferred correctly. Logos taken from UniPROBE database (13).
(TIF)

Table S1 **Results of each of the four methods on different reference motifs from the literature.** Each line gives the Euclidean distance between a PWM learned on PBM data and a PWM from another source. On the right-hand side, TOMTOM results are reported, giving the statistical significance of PWM similarity.
(XLS)

Table S2 **Results of each of the four methods on SCI09 PBM dataset for different positive probe set sizes (4sigma to 1sigma).** Each 2 consecutive lines refer to the paired PBM version of the same TF. The one listed under “PBM training data” is used for training, and the scores reported are for testing on the other one.
(XLS)

Table S3 **Results of each of the four methods on Harbison** ***et al.*** **dataset.** Each line gives the result of *in vivo* binding prediction on data taken from on experiment.
(XLS)

Methods S1 **Supplementary methods and results.**
(DOC)
