# Supplementary figures and images for: Assessment of Algorithms for Inferring Positional Weight Matrix Motifs of Transcription Factor Binding Sites Using Protein Binding Microarray Data
Source: PLoS One. 2012 Sep 28;7(9):e46145. doi: 10.1371/journal.pone.0046145 (PMC3460961; doi:10.1371/journal.pone.0046145)

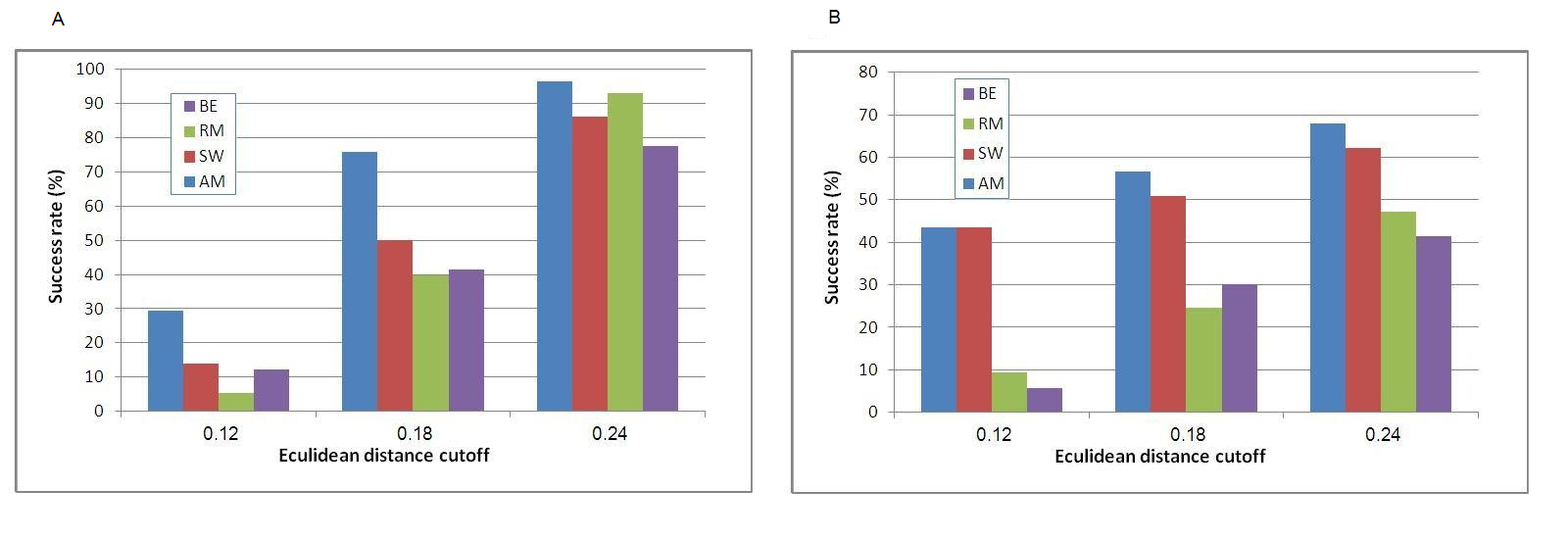

Supplement: Figure S1 — Similarity to experimentally established PWMs. (A) TRANSFAC motifs. For 80 proteins available in TRANSFAC we compared the motifs produced from their PBM data by each of the tested methods to the motif available in TRANSFAC. Distance was measured using Euclidean distance. Three distance cutoffs were used, 0.12, 0.18 and 0.24, and the fraction of recovered motifs with distance below the cutoff is the success rate. (B): ScerTF motifs. The same tests on 51 motifs from the ScerTF database. AM: Amadeus-PBM; SW: Seed&Wobble; RM: Rankmotif++; BE: BEEML-PBM. (TIF) [file pone.0046145.s001.tif]

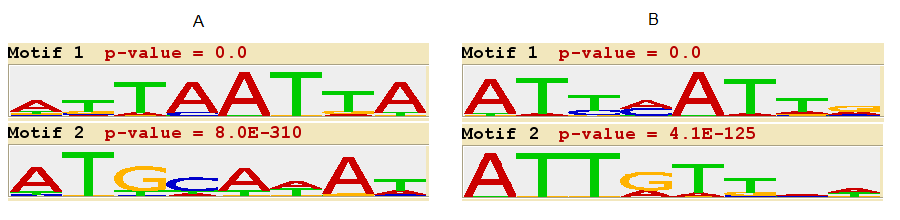

Supplement: Figure S2 — Shadow motifs. Examples of the primary and secondary motifs found by Amadeus for Pou2f3 (A) and Sox1 (B). p-values for the motif enrichment (hypergeometric score) are indicated above each motif. Note that even the second ranked motifs obtain extremely high significance. (TIF) [file pone.0046145.s002.tif]

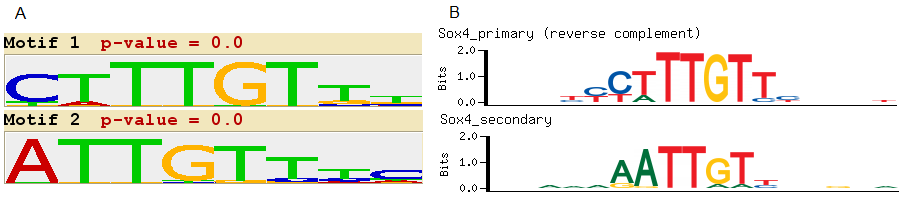

Supplement: Figure S3 — Sox4 primary and secondary motifs as found by Seed-and-Wobble (SW) and Amadeus-PBM (AM). Jauch et al. reported two motifs: CTTTGTT and AATTGTT (23). (A) The two top motifs recovered by AM. The first motif of Jauch et al. was recovered correctly; the second was partially recovered. (B) The two top motifs recovered by SW. Both motifs from Jauch et al. were inferred correctly. Logos taken from UniPROBE database (13). (TIF) [file pone.0046145.s003.tif]
