# Supplementary material for: Assessment of Algorithms for Inferring Positional Weight Matrix Motifs of Transcription Factor Binding Sites Using Protein Binding Microarray Data
Source: PLoS One. 2012 Sep 28;7(9):e46145. doi: 10.1371/journal.pone.0046145 (PMC3460961; doi:10.1371/journal.pone.0046145)
Supplement: Methods S1 — Supplementary methods and results. (DOC) [file pone.0046145.s007.doc]

**Supporting Information for**

Assessment of algorithms for inferring positional weight matrix motifs of transcription factor binding sites using protein binding microarray data

**by Orenstein, Linhart and Shamir**

**Supplementary Methods**

## Algorithms and parameters

BE, RM and SW were downloaded from the authors' websites and applied using the recommended parameters. The parameters for SW were k-mer length 8, seed pattern file patterns8of12, total pattern file patterns_4x44k_all_8mer. The parameters for RM were widths to try 7 to 13, no log transformation, optimize the scaling factor w, 400 negative sequences. The PWM with the best likelihood score was taken as the result. BE was run according to the R script provided on the authors' website. Its seed PWM was the SW result trained on the same array. Amadeus was run on the top 1000 9-mers as the target set, motif width 8 and all other parameters at default values. In particular, the background set included all 9-mers. For computing the Spearman rank coefficient, sensitivity at 1% false positive and AUC, the algorithms were run on one array and tested on the other. In the comparisons to known motifs and *in vivo* data, the algorithms were trained on both arrays together.

## Amadeus-PBM algorithm

We devised a simple scheme for detecting TFBS motifs in PBM data. The method is generic in that it can utilize any motif finding algorithm and any ranking score. Enrichment-based motif finding algorithms receive as input a target set of sequences that are expected to be enriched with the motif compared to other (background) sequences. Our approach to utilize such algorithm is quite simple:

1. Rank all *k*-mers according to some score that reflects their binding intensity.
2. Give the top *N* ranking *k*-mers as the target set to the motif finding algorithm, using all *k*-mers as the background set.

The rationale for using k-mers is that TFBSs have typically short motifs that will be reflected in overrepresented k-mers among high binding intensities. A PBM contains each 10-mer once, so that each non-palindromic 10-mer will appear twice, once on each strand. By choosing *k* < 10, each k-mer appears several times in the probe set, and the mean (or median) of the intensities of the probes containing these multiple occurrences can be used to rank the k-mers, thereby reducing noisy measurements and possible biases (due to, e.g., position in the probe sequence, flanking sequences and strand). We found the average binding intensity the most suitable. Note that the method works on the original binding intensities as reported in the PBM data. We found that using *k*=9 improved the accuracy of the results over *k*=8 when looking for a motif of length 8, since the set of top-ranking 9-mers may contain several shifts of the same 8-long motif (data not shown). Taking *N*=1000 proved to be a good compromise between adding more noise and leaving out too many 9-mers with true positive binding sites. Our motif finding algorithm of choice was Amadeus (15). We call the resulting method Amadeus-PBM.

## Mathematical criteria for evaluation of motif quality

**Position weight matrix (PWM):** The TFBS model used by all tested algorithms is a PWM: It is a *4×k* matrix *Θ*, where *Θ_i_(x)* is the probability of base *x* in a position *i* of the model. *k* varies with the motif. The score of k-mer w_1_...w_k_ is $\prod_{i=1}^{k} \Theta_{i}[w_{i}]$.

**Distance score:** To measure the distance between an inferred PWM to a reference PWM, we used *Euclidean distance* . The Euclidean distance of probability vectors *(v_1_, v_2_, v_3_, v_4_)* and *(u_1_, u_2_, u_3_, u_4_)* where ∑_i_ *v_i_* = 1 and ∑_i_ *u_i_* = 1 is:

$$d(u,v)=\sqrt{\sum_{i=1}^{4} (u_{i}-v_{i})^{2}}$$

If *p_1_* and *p_2_* are two aligned PWMs of length *k*, where *p_1i_* is the *i*-th column of *p_1_*, the Euclidean distance is

$$e(p_{1},p_{2})=\frac{1}{\sqrt{2}k}\sum_{i=1}^{k} d(p_{1i},p_{2i})$$

Hence, *0≤e≤1* with smaller values indicating higher similarity.

Given two PWMs, all possible (gap-free) alignments between them in both orientations with an overlap≥5 are tested, and the smallest obtained score is defined as the distance between the PWMs.

**Occupancy score:** Evaluating the chance that a PWM *Θ* binds to a probe or a promoter sequence *s* is done by summing the probabilities of all possible alignments of *Θ* to *s*. Formally, the *sum occupancy score* for sequence *s* and PWM *Θ* is defined as

$$f(s,\Theta)=\sum_{t=0}^{|s|-k} \prod_{i=1}^{k} \Theta_{i}[s_{t+i}]$$

Taking the sum was reported to give the better results than taking the maximum .

**Positive probes:** To evaluate how well a motif predicts the binding of probes in a PBM, one has to focus on the strongest, specific binding, as the lower binding intensities may be noisy and non-specific. Given the binding intensities of a protein to all probes in a PBM, Chen *et al*. defined the *positive probe set* as those probes whose normalized binding intensity is greater than the median by at least 4 * (MAD / 0.6745), where MAD is the median absolute deviation (MAD = 0.6745 for the normal distribution *N(0,1)*). We denote σ = (MAD / 0.6745). In some tests we also used larger positive probe sets by setting the threshold to 3σ, 2σ, and 1σ above the median.

**Positive promoters:** To evaluate how well a motif predicts the binding of a TF in a ChIP experiment, one has to identify the specific bindings. Haribson *et al*. defined the *positive promoter set* as those promoters whose reported p-value for binding to the TF is smaller than 0.001.

**Information content:** We used the entropy to measure the information content of each PWM . The bit information of vector *(v1, v2, v3, v4)* (where ∑_i_ v_i_ = 1) is defined as 2+i vi log(vi). The information content of a PWM is the average bit information of its columns.

**Ranking criteria:** To evaluate the probe (or sequence) ranking of an algorithm, we used the same three criteria as in . In all cases, we have *n* probes ranked x_1_≤x_2_≤…≤x_n_ according to some algorithm, while the true ranking according to binding intensities of probe *i* is *y_i_*. The **Spearman rank coefficient** compares how similar the two rankings of the positive probe set are, using the formula:

$$1-\frac{6\sum_{i=1}^{n} (x_{i}-y_{i})^{2}}{n(n^{2}-1)}$$

Suppose there are P positive and N negative probes. Denote z_i_=1 if probe *i* is in the positive set and z_i_=0 otherwise. If the algorithm assigns the top *t* probes as positive, there are $TP(t)=\sum_{j=1}^{t} z_{j}$ *true positive* and $FP(t)=t-TP(t)=\sum_{j=1}^{t} (1-z_{j})$ *false negative* samples. Of the remaining probes, which are declared negative, $FN(t)=P-TP(t)$ are *false negative* and $TN(t)=N-FP(t)$ are *true negative*. The *sensitivity* is defined as $TPR(t)=TP(t)/P$ and the *false positive rate* is$FP(t)/N$. Let *k* be the maximum number of top ranking probes that attain 1% false positive rate. Then the **sensitivity at 1% false positive** is defined as $TPR(k)$. The *receiver operating characteristic* (ROC) curve is defined as the function plotting the sensitivity against the false positive rate as *t* increases. The area under the ROC curve or **AUC** is used as the third criterion .

**Supplementary Results**

**Comparing predicted motifs to TRANSFAC and ScerTF**

We compared the motifs predicted by each method to PWMs reported in the TRANSFAC and ScerTF databases, in the same fashion as the comparison to JASPAR motifs. There were 80 TRANSFAC PWMs and 51 ScerTF PWMs corresponding to TFs from the SCI09 and GR09 studies, respectively, which did not originate from PBM data. Dissimilarity was measured using Euclidean distance. The results were clearly in favor of AM, followed by SW, RM and BE in this order (**Figure S1**). Full results are available in **Table S1**.

**Comparing the significance of the similarity to literature motifs**

As an additional quality measure, we scored the motif similarity using the recently developed Tomtom algorithm (24), which calculates the significance of the similarity. Given a query motif and a motif database, Tomtom outputs a p-value for the similarity of the query to different motifs in the database. AM showed the highest significance levels. For example, the number of PWMs with p-value below 0.01 threshold detected in the JASPAR database for AM, SW, RM and BE were 46, 40, 43 and 38, respectively. Results are available in **Table S1**.
